# The Algorithm of Determining an Anti-Collision Manoeuvre Trajectory Based on the Interpolation of Ship’s State Vector

**DOI:** 10.3390/s21165332

**Published:** 2021-08-06

**Authors:** Piotr Borkowski, Zbigniew Pietrzykowski, Janusz Magaj

**Affiliations:** Faculty of Computer Science and Telecommunications, Maritime University of Szczecin, Wały Chrobrego 1, 70500 Szczecin, Poland; z.pietrzykowski@am.szczecin.pl (Z.P.); j.magaj@am.szczecin.pl (J.M.)

**Keywords:** autonomous sea-going vessel, anti-collision manoeuvre, ship state vector, interpolation, Dijkstra’s algorithm

## Abstract

The determination of a ship’s safe trajectory in collision situations at sea is one of the basic functions in autonomous navigation of ships. While planning a collision avoiding manoeuvre in open waters, the navigator has to take into account the ships manoeuvrability and hydrometeorological conditions. To this end, the ship’s state vector is predicted—position coordinates, speed, heading, and other movement parameters—at fixed time intervals for different steering scenarios. One possible way to solve this problem is a method using the interpolation of the ship’s state vector based on the data from measurements conducted during the sea trials of the ship. This article presents the interpolating function within any convex quadrilateral with the nodes being its vertices. The proposed function interpolates the parameters of the ship’s state vector for the specified point of a plane, where the values in the interpolation nodes are data obtained from measurements performed during a series of turning circle tests, conducted for different starting conditions and various rudder settings. The proposed method of interpolation was used in the process of determining the anti-collision manoeuvre trajectory. The mechanism is based on the principles of a modified Dijkstra algorithm, in which the graph takes the form of a regular network of points. The transition between the graph vertices depends on the safe passing level of other objects and the degree of departure from the planned route. The determined shortest path between the starting vertex and the target vertex is the optimal solution for the discrete space of solutions. The algorithm for determining the trajectory of the anti-collision manoeuvre was implemented in autonomous sea-going vessel technology. This article presents the results of laboratory tests and tests conducted under quasi-real conditions using physical ship models. The experiments confirmed the effective operation of the developed algorithm of the determination of the anti-collision manoeuvre trajectory in the technological framework of autonomous ship navigation.

## 1. Introduction

The autonomy means of transport is one of the main directions of development in the transport industry. Today, unmanned aerial vehicles (UAVs) have become very popular and are increasingly used [1,2]. Similar to other modes of transport, maritime transport pays attention to remotely controlled and autonomous vehicles [3,4]. The reasons for doing research in this area include lowering the transport vehicle operational costs and minimizing human errors that result in accidents. This is possible by reducing the number of operators/crew members and by decreasing the scope of human decisions and actions. The research on autonomous transport vehicles, the drawing interest of manufacturers as well as transport operators, is conducted in many R&D centres. Due to its importance, the research is often co-financed by state administrations participating in projects, while in EU member states, the funding also comes from the central EU bodies. Examples are MONALISA 2.0 [5], MUNIN [6], STM [7], and AAWA [8]. The problem of safe route determination is one of the key issues that designers of autonomous ships have to cope with.

Considering various time spans, the determination of the ship’s track refers to the weather routing that accounts for changes in weather conditions during the voyage [9,10,11], strategic decisions or operational planning to prevent and avoid a collision [12,13,14,15], and operational decisions. In the former case, weather routing is mainly determined by the goals of the shipowner or charterer. Operational decisions are mainly affected by the prevailing navigational situation. In both cases, the factors to be considered are the ship’s manoeuvring capabilities and the hydrometeorological conditions. In particular, when a safe ship route, also known as a safe movement trajectory, is being determined in a collision situation, the short-term prediction of the ship movement parameters is required. Several factors should be taken into account. These are, besides the mentioned ship manoeuvring capabilities and hydrometeorological conditions, the present parameters of the ship’s state vector and the rudder and engine settings. This calls for the development of a ship dynamic movement model. The complexity of a ship’s movement process results from the impact of two environments: atmospheric (wind) and water (current, waves). The ship dynamic model is described by differential equations with different levels of detail. The more detailed the description, the more equations and higher computing complexity of the model has. This particularly applies to solving differential equations by numerical methods. The determination of a safe movement trajectory to avoid a collision with another ship requires the determination, verification, and comparison of many series of controls, thus the repeated solving of the same system of differential equations. This significantly extends the computing time, which may be acceptable in weather routing, but the long time needed to complete the calculations restricts its application in collision situations. The solution for a collision situation has to be found in the shortest time possible so that the ship is able to perform a proper and timely anti-collision manoeuvre. This also refers to simplifications, where the system of differential equations is replaced by a system of discrete differential equations.

An alternative solution is the estimation of the ship’s state vector on the basis of tables with the results of the ship’s sea manoeuvring trials. Due to the limited scope of data, as the number and types of manoeuvres and rudder/engine settings in sea trials are limited, the herein proposed solutions use a mathematical model of ship motion dynamics based on sea trial data. The model is used for generating the ship’s state vector parameters for the discrete values of the rudder and engine settings, taking into account the various starting conditions (the initial state vector values) and external factors (wind, waves). The model also requires the determination of the level and scope of discretization, including the discretization of the time and settings for the engine and the rudder. Regardless of the level and the scope of discretization, the ship’s state vector parameters have to be estimated, i.e., interpolated, to obtain intermediate values that have not been recorded during sea trials. The correct prediction of the ship’s state vector based on the estimated values of ship’s movement parameters is crucial when planning anti-collision manoeuvres.

The published methods of anti-collision manoeuvre determination are typically based on analytical models or artificial intelligence (AI) methods. In analytical methods, mathematical models with varying complexity levels are used [16,17,18,19]. In this case, challenges include the choice of the model and its structure and the identification of model parameters for the various navigational conditions. To determine the anti-collision manoeuvres, nowadays, AI methods are more frequently used, such as genetic and evolutionary algorithms [20,21,22,23,24], artificial neural networks [25,26,27], machine learning [28], or systems of fuzzy inference [29,30,31,32]. What restricts the use of these methods on an autonomous ship is essentially the lack of evidence on the stability of the solutions. This is important for the system to be reliable and for the assurance of the required level of navigational safety.

The major difficulty in both cases (analytical models and artificial intelligence) is to find a compromise between the details of reality description and computational complexity. The solution may be an approach using the ship’s state vector interpolation based on data obtained from measurements conducted during sea trials.

The article proposes an original algorithm for the determination of the trajectory of the anti-collision manoeuvre, which operates using the ship’s state vector interpolation. In order to reduce the computational complexity for the ship’s movement dynamics, tabular data recorded during sea trials were used. The modified Dijkstra’s algorithm [33] was used to determine the shortest path in the graph. The graph vertices represent the discretized space of the solutions in the form of regular grid of points. The transition between the graph vertices depends on the safety level of passing other vessels/objects and the degree of departure from the planned route. This requires the prediction of the ship’s movement parameters at the specific points of the plane: graph vertices. To achieve this, a function that performs interpolation within any convex quadrilateral with nodes as its vertices is proposed.

The algorithm described herein was implemented in an autonomous ship’s system and was verified in laboratory conditions and quasi-real conditions using physical ship models.

The further parts of the article are composed as follows. Section 2 proposes the interpolation method of the ship’s state vector. The algorithm for the determination of the anti-collision manoeuvre trajectory is presented in Section 3. Section 4 discusses the test results of the algorithm implemented in the anti-collision module of autonomous ship navigation. Section 5 summarizes the article.

## 2. Interpolation of Ship’s State Vector

**Figure 1:**
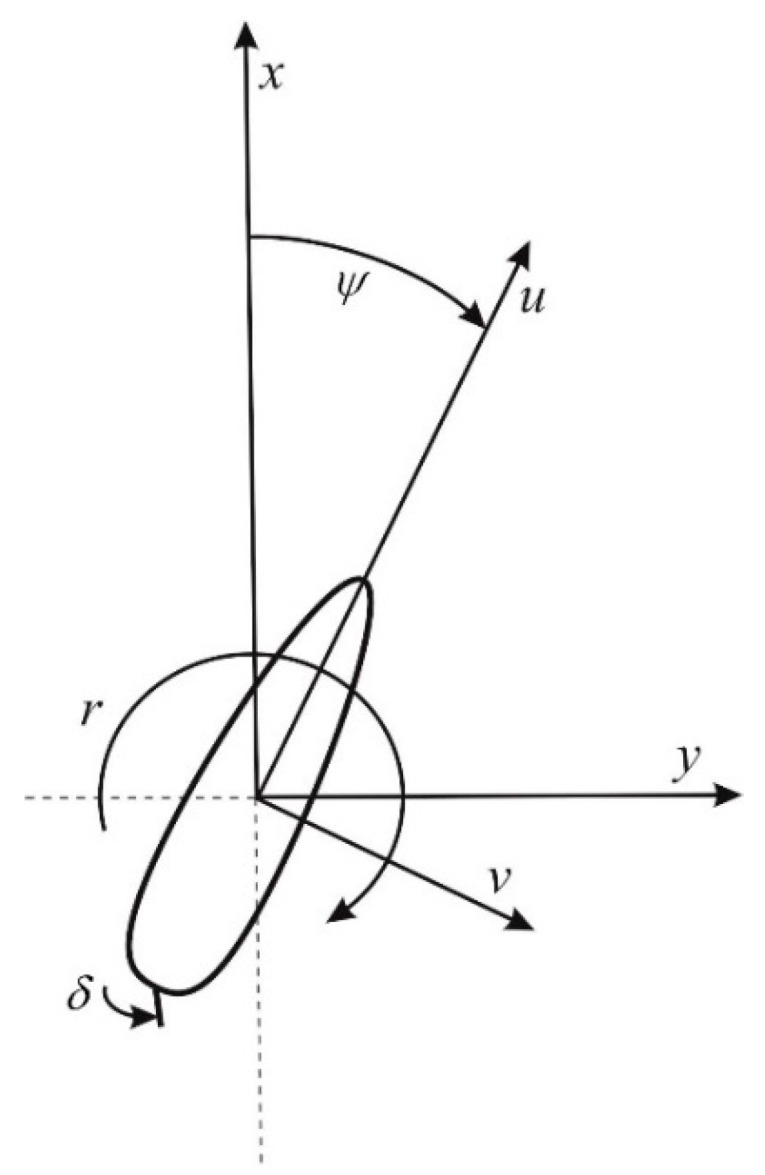
Quantities describing ship movement in the horizontal plane.

In the proposed approach, the ship’s state vector is defined as (Figure 1):

(1) [x,y,ψ,r,u,v]

where

(x,y)—Cartesian coordinates of ship position;
ψ—ship’s course;
*r*—rate of turn (angular speed);
*u*—longitudinal speed of the ship;
*v*—lateral speed of the ship.

The control signal is the rudder setting (δ). At present, one requirement for rudder drive design is that the power should be sufficient to ensure the change of rudder angle from $\delta_{min}$ to $\delta_{max}\;$takes no longer than 28 s (the average angular speed of the rudder is 2.33°/s). Taking the above into account, a simplification can be made:

(2) $$\delta =\delta_{current}$$

where

$\delta_{current}$ is the current rudder angle

In our approach, this should not lead to significant errors.

The mechanism of the movement state vector prediction applied in the algorithm to determine an anti-collision manoeuvre trajectory uses data obtained from measurements performed during a series of turning circle tests, conducted for different starting conditions and different rudder angles. Turning circle trials can be completed using real ships, a physical model of a ship, or a mathematical model of ship movement with arbitrarily selected complexity by taking into account the hydrometeorological conditions.

For a single trial of a turning circle manoeuvre established by meeting the starting conditions the following assumption can be made:

(3) $$x_{0}=0,\;\;y_{0}=0,\;\;r_{0}\in \langle r_{min},r_{max}\rangle ,\;\;\psi_{0}=0,\;\;u_{0}\neq 0,\;\;\delta_{0}=0$$

**Figure 2:**
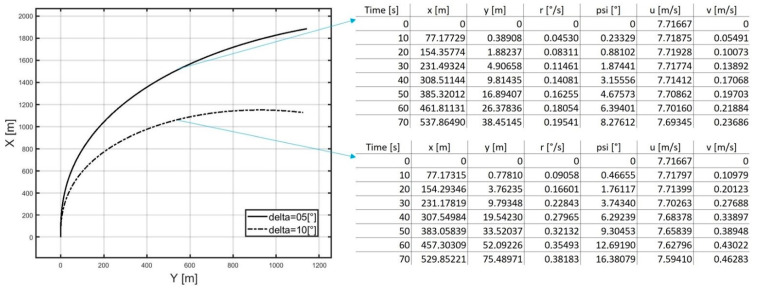
Example table fragments obtained from turning circle tests performed for the same starting conditions and varying rudder settings (δ=5°, δ=10°).

The rudder is set at a predetermined angle and remains in this position until the end of the trial. Additionally, the engine settings remain unchanged. During the trial, the changing values of the ship’s state vector parameters are recorded at arbitrarily chosen time intervals (for example, every 10 s) using available sensors. The recorded data can be stored in tables, with each table corresponding to one trial. Figure 2 shows an example image of table fragments obtained from turning circle tests performed for the same starting conditions and different rudder settings (δ=5°, δ=10°). For measurements of the ship’s position obtained using GNSS receivers (Global Navigation Satellite System) to conform with (1), the geographical position coordinates should be converted to the Cartesian coordinates using the the Gauss–Krüger mapping method [34]. The adopted origin of the Cartesian coordinate system is the point representing the ship’s centre of gravity at the instant that the turning circle test starts.

In the presented approach, so many turning circle trials have to be performed that in the selected hydrometeorological conditions and selected engine settings:

- Starting conditions should take into account the entire attainable range of the values of angular speed with the discretization step Δr selected to take account of $r_{0}=0\;\left(\left\{r_{min},r_{min}+\Delta r,r_{min}+2\Delta r,\ldots ,0,\ldots ,r_{max}\right\}\right)$;
- For the above starting conditions, the entire range of possible steering decisions should be taken into account with the discretization step Δδ selected to account for $\delta_{0}=0\;\left(\left\{\delta_{min},\delta_{min}+\Delta \delta ,\delta_{min}+2\Delta \delta ,\ldots ,0,\ldots ,\delta_{max}\right\}\right)$.

It is easy to notice that these conditions lead to an exponential increase in the number of tables. Hence, our approach is based on the assumption (2). For similar reasons, i.e., to reduce the size of the tables, the recording time of each turning circle test should be limited until the moment the ship’s angular speed becomes steady.

**Figure 3:**
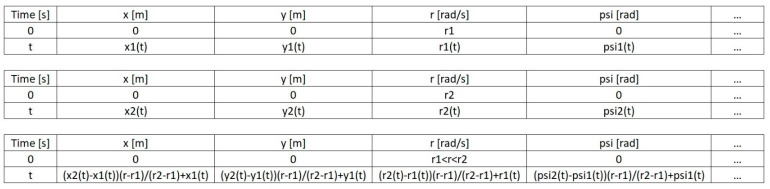
The method of determining (using linear interpolation) an example row of the table for the starting conditions corresponding to an intermediate value of angular speed *r* when the tables are given for the starting conditions corresponding to the angular speed values of *r*1 and *r*2 (r1<r<r2).

Having a set of tables, as described in the previous paragraph, and using the linear interpolation, we can determine the tables for the starting conditions corresponding to intermediate angular speed values or the intermediate rudder setting values. If the table for the starting conditions are to correspond simultaneously to both intermediate angular speed and rudder setting values, two-line interpolation should be used. Using linear interpolation, Figure 3 shows the method of determining an example row of the table for the starting conditions corresponding to an intermediate value of angular speed r when the tables are given for starting conditions corresponding to the angular speed values of *r*1 and *r*2 (r1<r<r2).

The discussed mechanism of linear and two-line interpolation (see Figure 3) provides the prediction of ship’s state vector for all steering scenarios at fixed time intervals and maintaining constant engine settings and steady hydrometeorological conditions. This is possible because by translating the vector opposite to the considered ship’s position and making a turn at an angle opposite to the considered ship’s course, the starting conditions in the determination of the future ship’s state vector will take the form conforming to the assumptions (3). Thus, using the appropriate tables, it is possible to determine the future value of the ship’s state vector, depending on the actual steering decision. This process will be able to be continued for a pre-set time span. Thus, a series of successive values of the ship’s state vector will be determined. If another prediction has to be made and if in the meantime, the hydrometeorological conditions change or the engine setting is altered, the table base will be replaced to suit the current situation.

**Figure 4:**
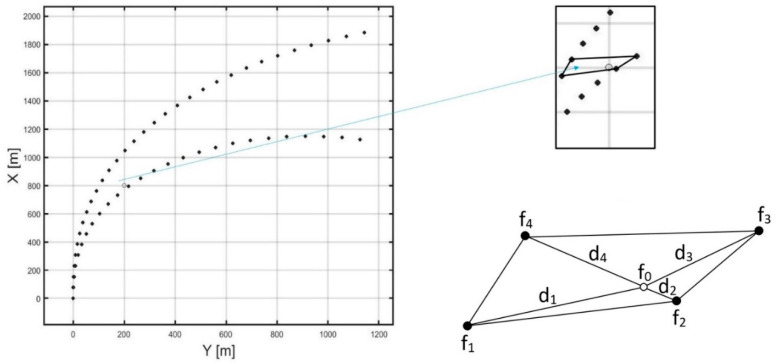
The visualized problem of the interpolation of the ship’s state vector for the specified point of the plane (the point for which the interpolation is to be made is marked with the tiny empty circle) where the measurements of the ship’s state vector are given at points resulting from the passages in each turning circle trial.

The proposed method of prediction allows for the determination of future values of the ship’s state vector, depending on the steering decision (rudder angle setting). For the purposes of the algorithm determining a trajectory of the anti-collision manoeuvre (described in the next section), it is necessary to predict the ship’s movement parameters for a specified point of a plane. The problem is illustrated in Figure 4. With the recorded values of the ship’s state vector at points resulting from ship passages in each turning circle test, the value of the state vector for the specified point of the plane should be determined (in Figure 4, an example point is marked with a tiny circle). The described problem comes down to constructing an interpolating function in the area of any convex quadrilateral whose vertices are the nodes. This condition disqualifies the use of two-line interpolation because for this operation, the nodes have to be the vertices of a rectangle. The looked-for function should be universal enough to correctly interpolate each of the parameters of the ship’s state vector by taking into account the location of the point relative to the interpolation nodes. Let

(4) $$f_{0}=\frac{w_{1}f_{1}+w_{2}f_{2}+w_{3}f_{3}+w_{4}f_{4}}{w_{1}+w_{2}+w_{3}+w_{4}}\;w_{1}=\frac{d_{2}+d_{3}+d_{4}}{d_{1}+d_{2}+d_{3}+d_{4}}\;w_{2}=\frac{d_{1}+d_{3}+d_{4}}{d_{1}+d_{2}+d_{3}+d_{4}}\;w_{3}=\frac{d_{1}+d_{2}+d_{4}}{d_{1}+d_{2}+d_{3}+d_{4}}\;w_{4}=\frac{d_{1}+d_{2}+d_{3}}{d_{1}+d_{2}+d_{3}+d_{4}}$$

where (Figure 4):

$f_{0}$—interpolated value of the function at the point lying inside the convex quadrilateral;
$f_{1},f_{2},f_{3},f_{4}$—the values of the function in the interpolation nodes (quadrilateral vertices);
$d_{1},d_{2},d_{3},d_{4}$—Euclidean distances of the point lying inside the quadrilateral from its vertices.

The proposed weighted average interpolates the values of the individual parameters of the ship’s state vector in such a way that the weight of the measurement becomes higher as the distance between the point set for interpolation, and the node becomes shorter. In the case illustrated in Figure 4, the highest value will be obtained by the weight $w_{2}\;$that corresponds to the measurement $f_{2}$.

The presented interpolation method of the ship’s state vector (4) may also be used to interpolate the time to reach a predefined point of the plane. This is possible because the tables contain the time of the individual measurements of the ship’s state vector.

To verify the correct operation of the proposed interpolation mechanism (4), 200 computational experiments were conducted for various starting conditions and rudder settings. In the simulations, the non-linear de Witt-Oppe model was used to represent a real object [35,36,37]. In all of the examined cases, the interpolation error did not exceed 10% of the difference between the maximum and the minimum value of the measurements in the interpolation nodes$\;\left(\left|max\left\{f_{1},f_{2},f_{3},f_{4}\right\}-min\left\{f_{1},f_{2},f_{3},f_{4}\right\}\right|\right)$. This error is acceptable for the algorithm determining trajectories of anti-collision manoeuvres.

## 3. The Algorithm of Determining Trajectories of an Anti-Collision Manoeuvre

As described in the previous section, the interpolation method of the ship’s state vector was used in the process of determining the trajectory of the anti-collision manoeuvre. This is a two-stage process. The first stage is the determination of the trajectory that is expected to resolve a collision situation (passing other objects). This stage is based on the principles of the modified Dijkstra algorithm [33] in which the graph takes the form of a regular grid of points. The transition between the graph vertices depends on the safety level of passing other objects and the degree of departure from the planned route. The determined shortest path between the starting vertex and the target vertex is the optimal solution for the discrete space of solutions. In the second stage, the vessel is supposed to return to its previously pre-set route. Schematically, the operation of the proposed algorithm for determining an anti-collision manoeuvre trajectory can be described in the following steps (stage 1 steps 1–5, stage 2–step 6):

1. Current assessment of the navigation situation;
2. Discretization of the pre-set space to determine the trajectory of the anti-collision manoeuvre, resulting in a graph or a regular grid of points (when a collision situation is detected);
3. Checking the availability of the graph vertices in the area of the steady rudder setting for the starting conditions corresponding to the current ship’s state vector;
4. Step 3 is to determine the subsequent area of the steady rudder setting in the starting conditions corresponding to the available lowest cost vertex from the previous area;
5. Step 4 should be performed until an area comprising an available vertex found in the last row of the grid is reached, while the starting conditions will be changed to those corresponding to the next available vertex of increasingly higher cost if there is no path leading to the target vertex;
6. Return to the set route (if there is a path leading to the target vertex).

Step 1

When the ship moves along the pre-set route, the navigational situation is continually assessed in relation to all other objects located within a set radius. The assessment of the navigation situation is based on the CPA (Closest Point of Approach) and TCPA (Time to Closest Point of Approach) [38,39,40]. Where the determined CPA value against any object happens to be less than the set safe CPA, and the corresponding value of TCPA will be positive (the ships have not passed each other yet), the situation will be then identified as a collision situation. This implies proceeding to step 2.

Step 2

**Figure 5:**
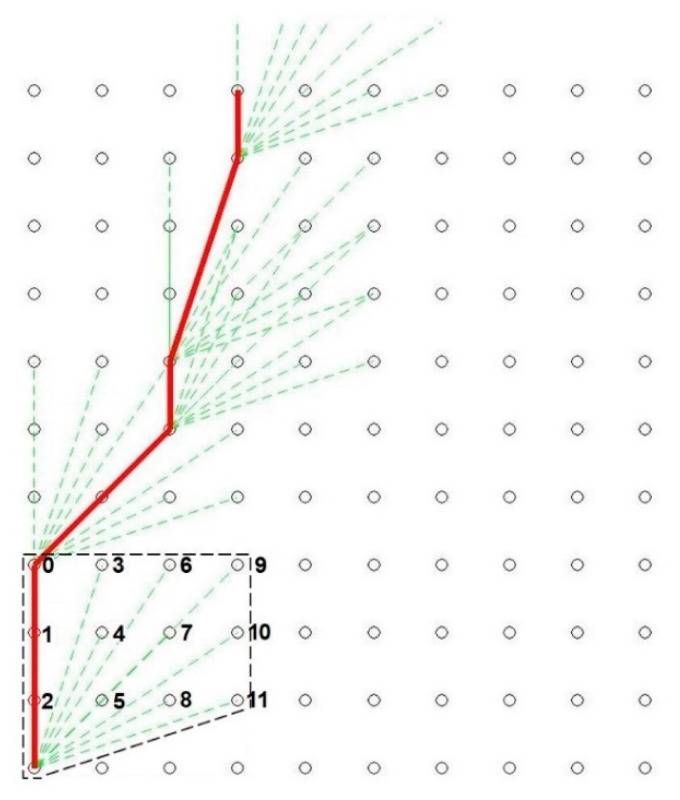
Visualized trajectory (determined in step 1) of an anti-collision manoeuvre in the discretized space and a marked area of steady rudder setting for starting conditions corresponding to the current ship’s state vector.

In Step 2, the set space for determining anti-collision manoeuvre trajectories is discretized to the form of a graph. In the case considered herein, the graph is in the form of a table with a set number of rows and columns. The distance between the vertices is constant and is defined as the grid (with the specified length and width). Both the size of its elements and of the entire grid are determined arbitrarily depending on the ship’s parameters and the implementation capabilities. The source (starting) vertex is located in the left bottom corner of the grid and corresponds to the current position of the ship, and the direction of the grid is consistent with the current ship’s course. Figure 5 shows an example of a discretized space for determining the trajectory of the anti-collision manoeuvre consisting of 11 rows and 10 columns.

There are connections from a selected graph vertex to other vertices that are only within the area of steady rudder setting. This area specifies the number of rows by which the ship can move upwards and the number of columns by which the ship can move to the right (to starboard) without changing the rudder setting. The area of the constant rudder angle is determined arbitrarily and unchanged for the entire process of trajectory determination for the anti-collision manoeuvre. In Figure 5, the black dashed line marks the area of the steady rudder setting for the starting conditions corresponding to the current ship’s state vector. Reaching the given vertex takes place at some cost, i.e., the deviation from the pre-set track and more frequent changes to the rudder settings. This cost is the lowest for the vertex located in the left upper corner—0, and is the highest for the vertex located in the right bottom corner—11, of the area under consideration.

Step 3

Step 3 checks the availability of the graph vertices in the area of the constant rudder setting for the starting conditions corresponding to the current ship’s state vector. There are two factors that decide whether a vertex is available. First, the possibility of reaching this vertex is determined from the tables (see Section 2) and is selected for the steering (rudder setting) limit decisions. Second, a vertex is not available if at the time it is reached, an excessive approach to any object occurs (the distance from another object turns out to be shorter than the safe pre-set CPA). This can be determined through the linear prediction of the other object’s position in the time equal to the time of reaching the examined vertex (time to reach the given vertex is calculated based on (4)).

Additionally, the availability of a given vertex may depend on its location on the appropriate side of the safety depth contour, which can be determined from data obtained from the ECS/ECDIS (Electronic Chart System/Electronic Chart Display and Information System). This is particularly important in cases where the applicability of the algorithm will be extended to include restricted areas.

Step 4

Step 4 is similar to performing Step 3 for the subsequent area of the steady rudder setting at starting conditions corresponding to the available lowest cost vertex from the previous area. The starting conditions are determined on the basis of Equation (4).

Step 5

Step 5 consists of performing Step 4 until the area containing the available vertex located on the last (highest) row of the grid is reached (target vertex; if a few vertices fulfil the defined condition, the vertex chosen as the one with lowest cost), while the starting conditions will be changed to those suiting the available vertex of increasingly higher cost in the case where no path leading to the target vertex exists. Step 5 can be successful, i.e., finding the shortest path (understood as the lowest total cost) leading to the final vertex. A trajectory that us developed in such a manner is shown as the red line in Figure 5. In the event of failure, the algorithm ends its operation, informing the operator that for the specified criteria, a solution cannot be determined. In this situation, the set safe CPA should be decreased.

Step 6

If at the end of Step 5 the shortest path is determined to be between the source vertex and the target vertex, then this path and the point marking the return to the original track will represent the trajectory of the anti-collision manoeuvre. The return to the set route is indicated by a point where the route intersects with the circle of the radius (the intersection point to be adopted is the one that is closer to the not yet reached point of turn of the set route):

(5) $$r=\frac{100+d_{1}}{100}d$$

where (Figure 6):

d—the distance between the source and the target vertices;
$d_{1}$—the indicator of track return smoothness (positive percentage value established arbitrarily: the longer the return, the higher the indicator is).

According to the Collision Regulations, to perform an anti-collision manoeuvre, it is recommended that the vessel turn to starboard (to the right). Such assumption is therefore adopted in the basic version of the algorithm. In the same way, an alternative solution can be determined (if it exists), which is composed of a series of turns to port (left). In this situation, i.e., when two possible trajectories are available, the solution that should be selected is the one burdened with the lower total cost associated with the deviation from the set route and an increase in number of rudder setting changes. This cost is calculated as the sum of the costs of reaching the individual vertices in the determined path.

When the algorithm is operating, its solution must be updated for the current input data with the maximum frequency possible to ensure effective operation.

## 4. Implementation of the Proposed Algorithm in the Framework of the Autonomous Ship Navigation Technology

**Figure 7:**
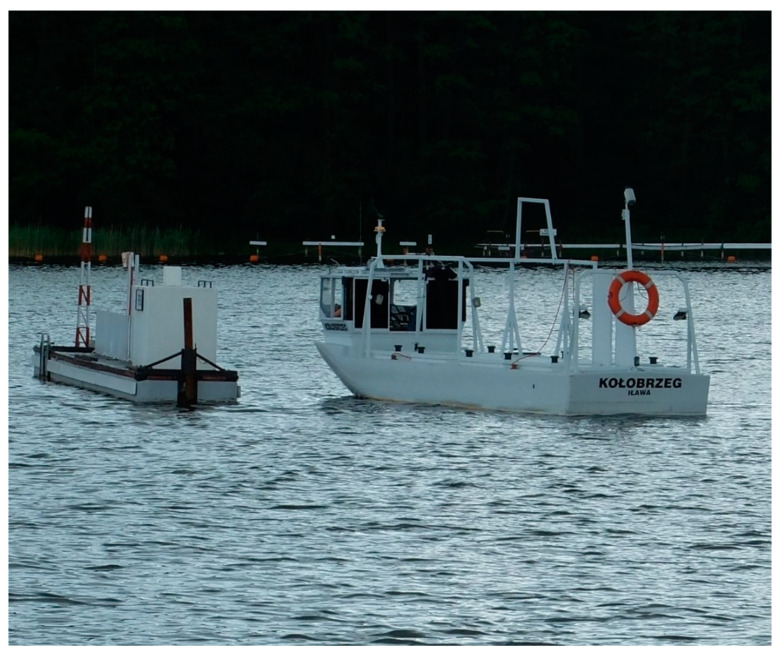
Physical model of the passenger ship Kołobrzeg used in the process of testing the AVAL technology.

The algorithm presented in the previous section was implemented in the anti-collision autonomous ship navigation module AVAL [41]. The AVAL technology features functionalities of fully autonomous navigation and the control of unmanned sea-going vessels along with the automatic communication service, assuring negotiations during the conduct of anti-collision manoeuvres. In addition, AVAL provides integration with unmanned aerial vehicles, enabling the acquisition of data on objects not detected by standard navigation systems (Automatic Identification System, Automatic Radar Plotting Aids). The AVAL technology was tested in laboratory conditions and then in quasi-real conditions using physical ship models (Figure 7). Computational experiments conducted in laboratory conditions were based on a model of 3DOF (degrees of freedom) in the following form (equations describing the forces and moments and the coefficients used are taken from [42,43,44]):

(6) $$\dot{x}=u\cdot cos\left(\psi \right)-v\cdot sin\left(\psi \right)\;\dot{y}=u\cdot sin\left(\psi \right)+v\cdot cos\left(\psi \right)\;\dot{\psi }=r\;\dot{u}=\left(X_{k}+X_{r}+X_{p}+X_{a}\right)/m+v\cdot r\;\dot{v}=\left(X_{k}+X_{r}+X_{p}+X_{a}\right)/m+u\cdot r\;\dot{r}=\left(N_{k}+N_{r}+N_{p}+N_{a}\right)/I_{z}$$

where

$X_{k},Y_{k},N_{k}$—force (longitudinal and transverse components) and the moment of resistance of water affecting the underwater part of the hull;
$X_{r},Y_{r},N_{r}$—force (longitudinal and transverse components) and the moment of the rudder resistance (effective rudder angle and rudder surface area);
$X_{p},Y_{p},N_{p}$—force (longitudinal and transverse components) and the moment of resistance of the propeller (diameter and rpm of the propeller);
$X_{k},Y_{k},N_{k}$—force (longitudinal and transverse components) and the moment of resistance of the external conditions (wind, waves, current);
$I_{z}$—ship’s moment of ship;
m—mass of the ship.

The verification of the anti-collision AVAL technology module was to verify whether the implemented algorithm operates correctly in determining the trajectory of an anti-collision manoeuvre. The ability to avoid a collision with moving objects was verified. This requirement is considered to be met when a safe distance that is the closest point of approach is maintained for all targets during an experiment.

**Figure 8:**
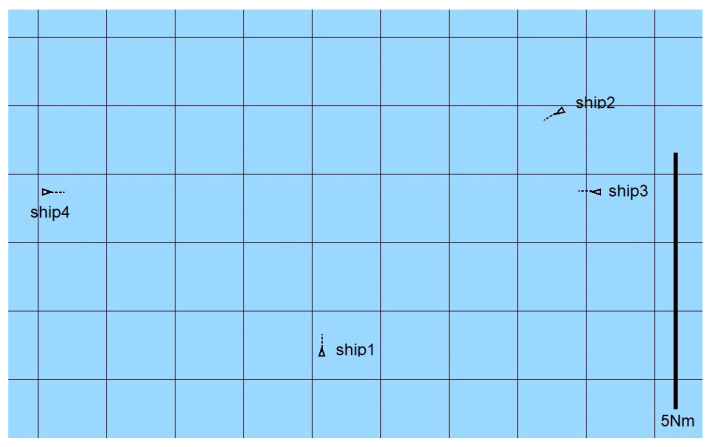
Scenario of a collision situation encountered by four ships—Example 1.

**Figure 9:**
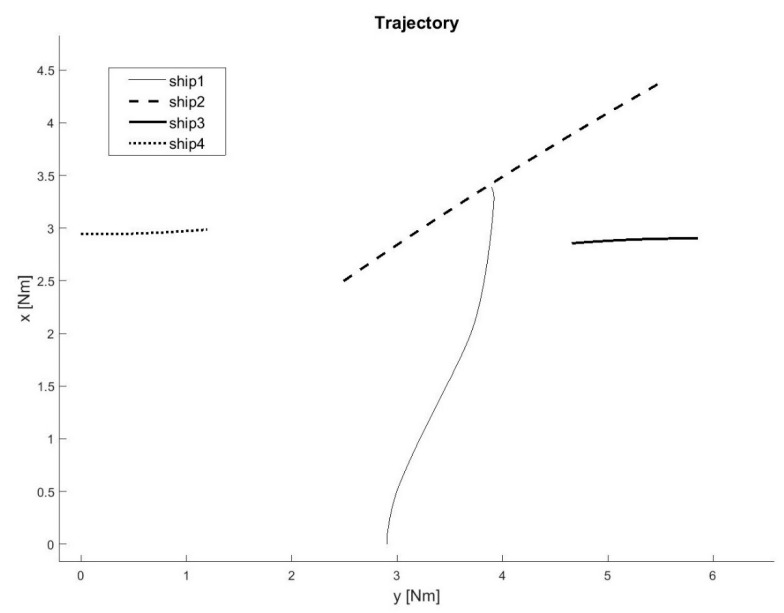
Execution of Scenario 1 with the anti-collision manoeuvre worked out by Ship 1—ship movement trajectories.

**Figure 10:**
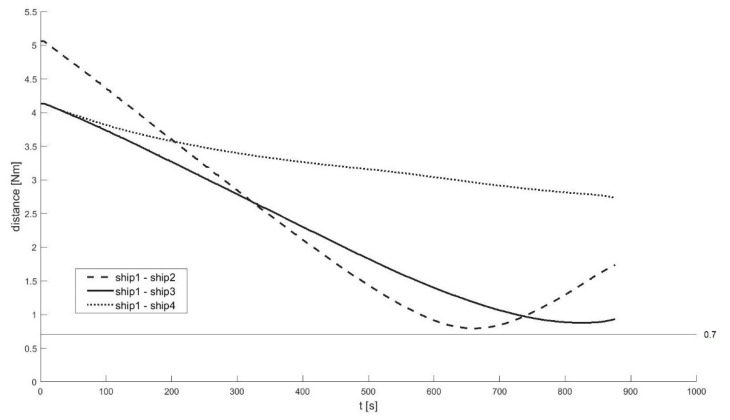
Execution of Scenario 1 with the anti-collision manoeuvre developed by Ship 1—Distances between the ships.

**Figure 11:**
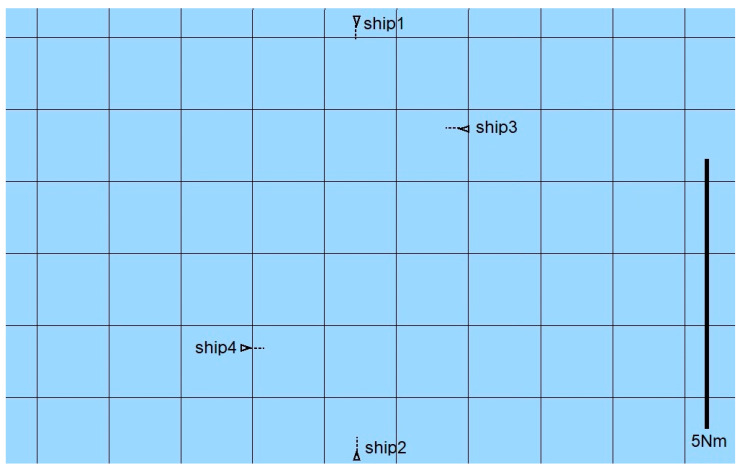
Scenario of a collision situation at encountered by four ships—Example 2.

**Figure 12:**
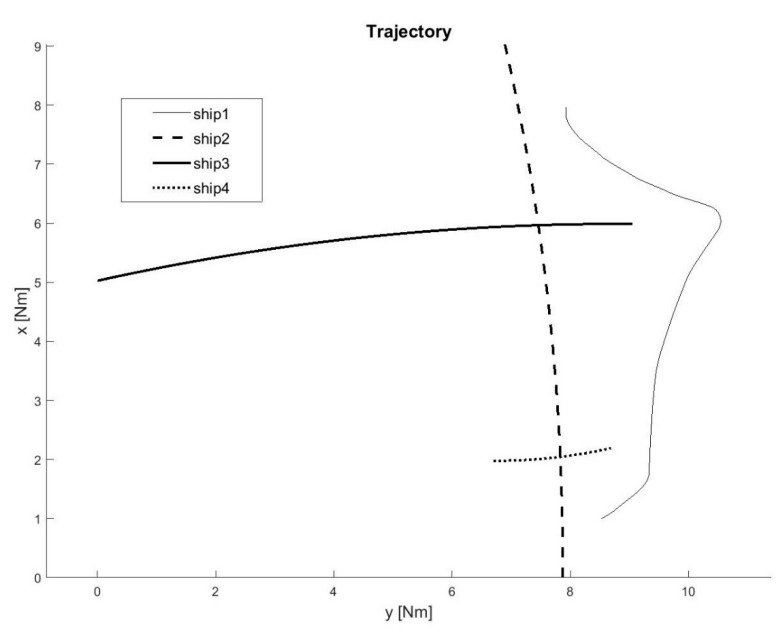
Execution of Scenario 2 with the anti-collision manoeuvre worked out by Ship 1—ship movement trajectories.

**Figure 13:**
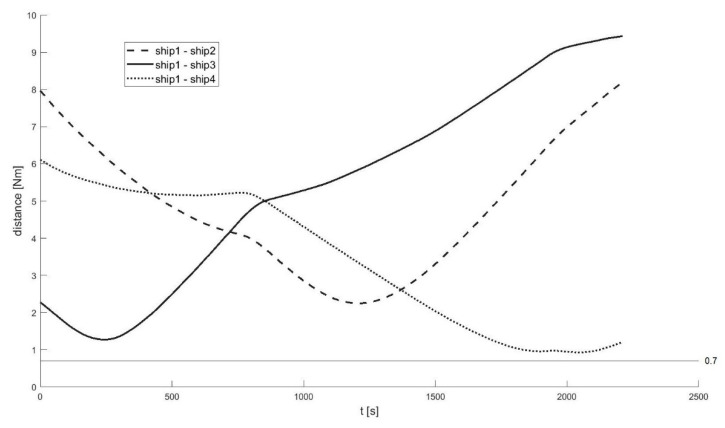
Execution of Scenario 2 with the anti-collision manoeuvre developed by ship 1—Distances between the ships.

A total of 60 ship passages were executed altogether (30 simulated passages in the lab and 30 passages in quasi-real conditions, using physical ship models at the Ship Handling Research and Training Centre in Iława [45]), by implementing various scenarios of ships meeting on collision courses (Figure 8, Figure 9, Figure 10, Figure 11, Figure 12 and Figure 13). Figure 8 and Figure 11 present two examples of collision situations (encountered by four ships; Ship 1 is expected to work out an anti-collision manoeuvre). Figure 9 and Figure 12 illustrate the execution of these scenarios, i.e., the trajectories of the ships. In addition, Figure 10 and Figure 13 present the time-varying plots of the distance between the ships. It was assumed in the tests that the safest and closest point of approach was 0.7 Nm. In the execution of Scenario execution, the actual distance (CPA) was 0.79 Nm (to Ship 2), 0.88 Nm (to Ship 3), 2.62 Nm (to Ship 4). In Scenario 2, the actual CPA was 2.25 Nm (Ship 2), 1.27 Nm (Ship 3), 0.93 Nm (Ship 4). In both experiments, the assumed minimum CPA was maintained in relation to all objects in the vicinity.

The presented results are typical of the performed tests. In all of the analysed cases, the AVAL technology performed its anti-collision function correctly. The ship passages confirmed that the assumed closest point of approach was maintained. The trajectories of the anti-collision manoeuvres generated by the system were also positively assessed by the expert navigators.

Currently, there are no available tools featuring the function of automatic determination for anti-collision manoeuvre trajectory. Therefore, it was not possible to compare the performance of the proposed algorithm with alternative solutions.

## 5. Conclusions

This article proposes an original algorithm to determine a ship’s movement trajectory in a collision situation. The solution is determined on the basis of tabular data obtained from measurements conducted during sea trials. Due to the discretization of the solution space, it was necessary to develop an effective and rapid method of interpolation for the ship’s state vector for the specified point of the plane. To achieve this, a function has been proposed that performs interpolation within any convex quadrilateral with the nodes being its vertices. This allowed the reduction of the restrictions resulting from the complexity of the calculations compared to classical tools. At the same time, the algorithm guarantees the correct solution (modified Dijkstra’s algorithm), which is not always the case when artificial intelligence methods are used.

The algorithm was implemented in the system of an autonomous ship and was then verified in laboratory conditions and quasi-real conditions using physical ship models. The experiments confirmed the effective operation of the algorithm for the determination of the anti-collision manoeuvre trajectory, which is part of technology of autonomous ship navigation. The applicability of the presented solution in developing the autonomy of transport vehicles fits into the field of modern maritime navigation [46,47,48,49,50,51,52].

Future directions for research on the proposed algorithm for determining an anti-collision manoeuvre trajectory include:

- Broadening the applicability of the algorithm to include restricted areas (stage 2, i.e., the return to the set route, since stage 1 takes into account both static and dynamic restrictions);
- Automation of the selection of total grid size and the size of its elements; the grid is a discrete space for determining the anti-collision manoeuvre trajectory;
- Examination of the feasibility of adding such steps as combined turns to the right and left(starboard and port) to the algorithm.

## Figures and Tables

**Figure 6:**
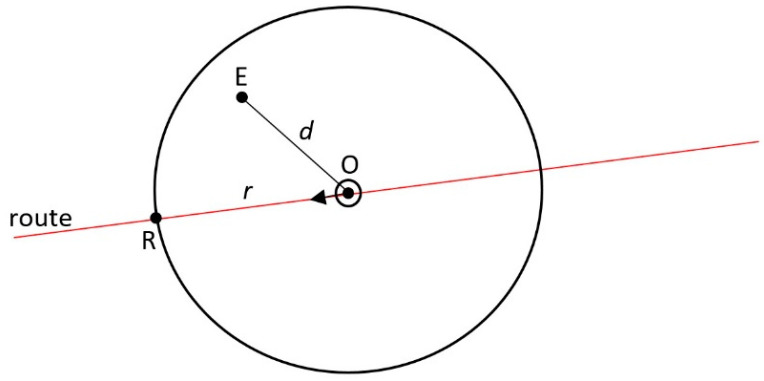
Return to the set route (O—point indicating the current position of the ship, E—point corresponding to the target vertex, R—point defining the return to the set route).

## Data Availability

Not applicable.
